# Alternative Transcript Initiation and Splicing as a Response to DNA Damage

**DOI:** 10.1371/journal.pone.0025758

**Published:** 2011-10-19

**Authors:** Carl N. Sprung, Jason Li, Daniel Hovan, Michael J. McKay, Helen B. Forrester

**Affiliations:** 1 Centre for Innate Immunity and Infectious Disease, Monash Institute for Medical Research, Monash University, Clayton, Victoria, Australia; 2 Peter MacCallum Cancer Centre, Melbourne, Victoria, Australia; 3 Department of Radiation Oncology, Canberra Hospital, Garran, Australian Capital Territory, Australia; 4 The Australian National University, Canberra, Australian Capital Territory, Australia; National Cancer Institute, United States of America

## Abstract

Humans are exposed to the DNA damaging agent, ionizing radiation (IR), from background radiation, medical treatments, occupational and accidental exposures. IR causes changes in transcription, but little is known about alternative transcription in response to IR on a genome-wide basis. These investigations examine the response to IR at the exon level in human cells, using exon arrays to comprehensively characterize radiation-induced transcriptional expression products. Previously uncharacterized alternative transcripts that preferentially occur following IR exposure have been discovered. A large number of genes showed alternative transcription initiation as a response to IR. Dose-response and time course kinetics have also been characterized. Interestingly, most genes showing alternative transcript induction maintained these isoforms over the dose range and times tested. Finally, clusters of co-ordinately up- and down-regulated radiation response genes were identified at specific chromosomal loci. These data provide the first genome-wide view of the transcriptional response to ionizing radiation at the exon level. This study provides novel insights into alternative transcripts as a mechanism for response to DNA damage and cell stress responses in general.

## Introduction

The transcriptional response to cellular stress is critical for cell survival. Ionizing radiation (IR) causes a broad spectrum of DNA damage for which the cells' commitment to repair, programmed death, cell division arrest or senescence, is required for an organism's survival. Humans are ubiquitously exposed to radiation, including during cancer treatment. About 1–5% of radiotherapy patients have severe side effects. The rate of these normal tissue reactions, for which there is nearly a normal distribution across the population, is determined by a therapeutic ratio (tumour control/adverse normal tissue reactions). The outcome of this radiation response diversity is that the dosage all patients receive is limited by those few patients who are particularly radiosensitive in their normal tissue, and thereby ultimately preclude optimal treatment for the majority of radiotherapy patients. Identification of those patients who are radiosensitive given current dose regimes, is paramount to enable individualization of RT. These individuals can potentially be identified by interrogating the transcriptome. Furthermore, identifying the IR transcriptional response profile also benefits establishment of biological dosage predictors, understanding response to other radiological exposures and can contribute to the development of new radio-pharmaceuticals.

DNA damage is a major cellular consequence upon exposure to radiation, where double-strand breaks (DSBs) are a critical type of damage that can lead to cell death and potentiate tumorigenesis. Two major pathways involved in DNA DSB repair are non-homologous end-joining and homologous recombination both involving many proteins [1]. Proper repair of DNA DSBs requires accurate damage recognition and signalling to initiate a cell cycle block to allow time to complete the DNA DSB repair process [2]. When DNA damage exceeds tolerable amounts, a cell may initiate signalling cascades leading to apoptosis, autophagy, necrosis or senescence. Hence, in response to IR, the expression of a variety of different types of genes are required.

The IR response at the transcriptional level has been characterized to some degree in different experimental settings for the human genome [3], [4], [5], [6], [7]. Additionally, whole genome analysis of IR transcriptional responses using platforms which target the 3′ end of transcripts, has been completed in a number of cell types including lymphoblasts [4] and fibroblasts [5]. However, to date, no study has completed a whole genome analysis for the response to radiation comprehensively at the exon level.

Post-transcriptional processing is a primary mechanism to generate protein diversity. In particular, the production of alternative transcripts leading to multiple isoforms is common for many genes. Different transcription isoforms can result in dramatically different cellular responses. Use of alternative transcripts is predicted for the majority of human genes [8], [9]. Sequencing of B cells for example yielded 94% of multi-exon genes had alternative transcripts [10]. Loss of protein functional domains due to alternative splicing (AS) is common and can have profound effects on function [11]. Alternative transcription products can also cause transcript instability [12], [13].

Alternative transcripts can be grouped into different categories. These include alternative use of exons, different 5′ and 3′ splice sites, alternative transcription start sites, alternative termination sites, as well as intron retention and mutually exclusive alternative exons [14]. The addition or loss of a complete exon has been suggested to account for a third of alternative transcription products whereas about a quarter are proposed to be due to alternative selection of 3′ and 5′ splice sites [14]. Loss of RNA sequence that code for a functional domain can directly affect protein function, and can lead to production of dominant negatives in some cases. Also, loss of signalling sequences may result in faulty localization of a protein. Loss of a regulatory domain may lead to loss of function or may antagonize function as observed with Bcl [15]. Alteration of the 5′ untranslated region of the transcript can have a profound effect on overall gene function. For example, spliced out localization signals can result in altered protein localization [13], [16]. Furthermore, it is reported that one third of all alternative transcripts are truncated, which commonly results in activation of the nonsense mediated decay pathway leading to decreased transcript levels and is a major regulator of protein production [17].

Alternative transcription is important in the regulation of genes involved in many cell processes and genetic diseases including cancer [18], [19], [20], [21], [22], [23]. Common in cancer cell lines, shorter RNA isoforms due to alternative polyadenylation sites often have increased protein levels which in some cases is due to the loss of microRNA-mediated repression [24].

Inhibition of RNA polymerase II elongation has been shown to be a mechanism for genotoxic stress (ultraviolet radiation) induction of AS proposed to be due to allowance of weaker splicing sites to participate in AS [25]. AS, in response to IR, has also been reported for a few genes. For example, in *Drosophila melanogaster*, the TAF1 gene (a subunit of TFIID involved in RNA polymerase II transcription), is alternatively spliced following IR [26]. In mammalian cells, clusterin has a complex response to IR, and in part, involves AS resulting in a isoform that is cytoprotective after IR [27]. Furthermore, ATF3, a transcription factor that is induced in response to IR [28], [29], has two main AS products, one isoform lacking the leucine zipper domain resulting in opposing activity in response to stress [30], [31], [32]. A splice variant of nucleophosmin, when over-expressed causes cell survival increase following IR in HeLa cells [33]. *RAD17*, involved in cell cycle arrest, is another example of a gene that is alternatively spliced in response to radiation [34]. However, no study to date has reported AS on a genome-wide scale in response to IR.

Some specific genes have been found to use a secondary promoter following IR to produce a radiation-induced isoform. These genes include *MDM2* [35], *PPM1D* [36] and *FBXW7* [37]. The whole genome investigations presented in this report show that many genes feature this specific response to IR.

Human exon arrays were used to identify transcription changes, including the induction of AS, in cells exposed to IR. Unlike some other oligonucleotide array platforms that have probe sets only at the 3′ end of the transcripts, the exon array has an average of nearly four probe sets for every known exon. This allows the determination of relative levels of each exon for a given treatment, facilitating identification of exons that are differentially expressed after IR. Having a transcript profile for every exon has the advantage of being able to detect transcripts which classical 3′ assay platforms would miss. For example, exon arrays are able to detect transcripts missing the 3′ exon for a number of reasons such as degradation, splicing, or undefined 3′ ends. Transcripts with non-polyadenylated messages or alternative polyandenylation sites would also be commonly missed. Given present estimates that most genes can use AS [8], it is apparent that AS is an important aspect of profiling expression. It is also clear that post-transcriptional regulation has a profound influence on the overall regulation of the proteome, including the response to IR and other genotoxic agents.

Here we analyse the human transcriptome IR response for both dose and time in two human cell types, lymphoblastoid cell lines (LCLs) and primary fibroblasts at the level of individual exons. Alternative transcription and other genome-wide transcription features have been identified as a response to IR.

## Results

### Global radiation response

Whole genome transcript exon arrays were utilized to comprehensively characterize radiation-induced transcriptional expression products in two human cell types. The exon array is set up with 4 probes for most probe selection regions (PSRs), and represents an exon or potential exonic region of a gene. For these studies, the filtered ‘core’ set of PSRs (RefSeq transcripts and full length mRNAs; Affymetrix.com) which are well-documented exon regions, were utilized. RNA processed from human lymphoblast and fibroblast cell lines exposed or sham-exposed to radiation was run on exon arrays to examine the transcriptional profile in response to radiation at the exon level. The extensive transcript coverage and relatively large number of samples allowed us to obtain robust whole gene expression levels inclusive of exon specific expression for LCLs and primary fibroblasts in response to 10 Gy at 4 hours post-IR. Therefore, the majority of the analyses were performed on data from this dose and time. Similar conditions have been used in previous IR response papers [3], [4]. Using RMA normalization (background correction) and SAM analysis [38], we identified genes modulated in response to IR (Tables 1, 2; S2 and S3). Eleven of the top 20 up-regulated genes and four of the top 20 down-regulated genes were the same for both LCLs and fibroblasts (Tables 1 and 2). Many of the identified genes have previously been observed to be modulated following IR. *CDKN1A*, *MDM2*, *PPM1D*, *GADD45A*, *SESN2*, *CCNG1* and *XPC* [3], [4], [5], [6], [39], [40] are examples which act as known controls and behaved as expected. The exon arrays also enabled the identification of genes, not previously reported to be statistically significantly modulated after IR in human LCLs (Table S4; e. g., from Table 1: *EDA2R*, *FAM72A* and *C1orf183*) or in fibroblasts (Table S5; e. g., from Table 2: *ASAH3L* (*ACER1*), *EDA2R*, *PAG1*, *BCOR*, *CBL*, *FAM100B*, *FAM72A*, *SETD8* and *TIGD1*), although some of these genes are IR-responsive in other experimental settings.

**Table 1:** Top genes modulated in LCLs 4 hours following 10 Gy of IR.

Up-regulation				Down-regulation			
Gene Sym.	GenBank Acc.	Fold-Change	Adjusted P-Value†	Gene Sym.	GenBank Acc.	Fold-Change	Adjusted P-Value†
*BLOC1S2*	NM_001001342	1.75	<0.01	*ARHGAP11A*	NM_014783	−1.99	<0.01
*C12orf5*	NM_020375	2.37	<0.01	*ASPM*	NM_018136	−2.58	<0.01
*C1orf183*	NM_019099	2.46	<0.01	*AURKA* *	NM_198433	−2.73	<0.01
*CDKN1A* *	NM_078467	2.94	<0.01	*BUB1*	NM_004336	−2.05	<0.01
*EDA2R* *	NM_021783	2.67	<0.01	*CCNB1*	NM_031966	−2.93	<0.01
*EI24*	NM_004879	1.66	<0.01	*CDC20*	NM_001255	−3.13	<0.01
*FAS*	NM_000043	1.78	<0.01	*CENPA* *	NM_001809	−2.17	<0.01
*FBXO22*	NM_147188	1.87	<0.01	*CENPE*	NM_001813	−2.98	<0.01
*GADD45A* *	NM_001924	1.98	<0.01	*DEPDC1*	NM_001114120	−3.03	<0.01
*GDF15* *	NM_004864	2.87	<0.01	*DLG7*	NM_014750	−2.68	<0.01
*ISG20L1*	NM_022767	1.75	<0.01	*FAM72A* *	BC035696	−2.81	<0.01
*MDM2* *	NM_002392	2.22	<0.01	*GTSE1*	NM_016426	−1.85	<0.01
*PHLDA3*	NM_012396	2.62	<0.01	*INCENP*	NM_001040694	−1.38	<0.01
*PLK2*	NM_006622	3.79	<0.01	*KIF20A*	NM_005733	−4.63	<0.01
*POLH* *	NM_006502	2.17	<0.01	*KIF23*	NM_138555	−2.21	<0.01
*PPM1D* *	NM_003620	2.53	<0.01	*NEK2*	NM_002497	−1.92	<0.01
*SESN2* *	NM_031459	2.08	<0.01	*PLK1* *	NM_005030	−4.17	<0.01
*TNFRSF10B* *	NM_003842	1.72	<0.01	*TACC3*	NM_006342	−1.67	<0.01
*XPC* *	NM_004628	2.03	<0.01	*TPX2*	NM_012112	−1.92	<0.01
*ZNF79* *	NM_007135	1.91	<0.01	*UBE2C*	NM_181802	−1.72	<0.01

*Genes that are also found in the top fibroblast cells gene list (Table 2).

† Exact p-values and adjusted p-values are provided in supplemental materials.

**Table 2 pone-0025758-t002:** Top genes modulated in fibroblast cells 4 hours following 10 Gy of IR.

Up-regulation				Down-regulation			
Gene Sym.	GenBank Acc.	Fold-Change	Adjusted P-Value†	Gene Sym.	GenBank Acc.	Fold-Change	Adjusted P-Value†
*ASAH3L (ACER1)*	NM_001010887	1.54	<0.01	*AURKA* *	NM_198433	−2.63	<0.01
*BTG2*	NM_006763	2.6	<0.01	*BCOR*	NM_001123385	−1.24	0.016
*CDKN1A* *	NM_078467	2.63	<0.01	*C13orf34*	NM_024808	−2.26	<0.01
*DDB2*	NM_000107	1.49	<0.01	*CBL*	NM_005188	−1.2	0.04
*EDA2R* *	NM_021783	1.57	<0.01	*CCNF*	NM_001761	−1.79	<0.01
*GADD45A* *	NM_001924	1.77	<0.01	*CDCA8*	NM_018101	−1.96	<0.01
*GDF15* *	NM_004864	2.39	<0.01	*CENPA* *	NM_001809	−1.61	0.02
*MDM2* *	NM_002392	2.27	<0.01	*CKS2*	NM_001827	−1.8	0.028
*PAG1*	NM_018440	1.57	<0.01	*FAM100B*	BC035511	−1.24	0.026
*PLK3*	NM_004073	1.69	<0.01	*FAM72A* *	BC035696	−1.64	0.036
*POLH* *	NM_006502	1.56	<0.01	*FAM83D*	NM_030919	−1.86	<0.01
*PPM1D* *	NM_003620	1.75	<0.01	*GAS2L3*	NM_174942	−2.2	<0.01
*RNF19B*	NM_153341	1.51	<0.01	*HJURP*	NM_018410	−1.98	0.011
*SESN1*	NM_014454	2.17	<0.01	*KIAA1333 (G2E3)*	NM_017769	−1.86	0.013
*SESN2* *	NM_031459	1.6	<0.01	*KIF18A*	NM_031217	−2.4	0.015
*TNFRSF10B* *	NM_003842	1.58	<0.01	*KLF12*	NM_007249	−1.24	0.034
*TNFRSF10C*	NM_003841	1.64	<0.01	*PLK1* *	NM_005030	−2.23	0.015
*TP53INP1*	NM_033285	1.99	<0.01	*PSRC1*	NM_001032290	−1.42	0.037
*XPC* *	NM_004628	1.39	<0.01	*SETD8*	NM_020382	−1.19	0.022
*ZNF79* *	NM_007135	1.36	<0.01	*TIGD1*	NM_145702	−1.24	0.039

*Genes that are also found in the top LCL gene list (Table 1).

† Exact p-values and adjusted p-values are provided in supplemental materials.

### Alternative transcripts

The differences in PSR transcripts four hours after exposure to IR for all genes were determined using an AS ANOVA (Partek Genomics Suite) and FIRMA and calculated splicing indexes for each exon. We have identified many genes that show different PSR expression changes within the gene in LCLs (Tables 3 and S6) and fibroblasts (Tables 4 and S7). Approximately half of the genes on these lists for both up- and down-regulated genes are present in both the LCL and fibroblast cell lists. Nine of the top 20 up-regulated genes for both LCLs and fibroblasts are also present in the top genes for alternative splicing (Tables 1–4). 13 and 12 of the top 20 down-regulated genes for LCLs (Table 1) and fibroblasts (Table 2) cells, respectively, were also found to be the top alternatively spliced gene (Tables 3 and 4).

**Table 3 pone-0025758-t003:** Genes predicted to produce alternative transcripts in LCLs 4 hours following 10 Gy of IR.

Up-regulation				Down-regulation			
Gene Sym.	GenBank Acc.	Partek Alt Splice P-Value	FIRMA Alt Splice P-Value	Gene Sym.	GenBank Acc.	Partek Alt Splice P-Value	FIRMA Alt Splice P-Value
*ASTN2*	NM_198186	<0.0001#	0.0002	*ANLN*	NM_018685	<0.0001#	0.0004
*BBC3*	NM_001127240	<0.0001#	<0.0001#	*AURKA* *	NM_198433	<0.0001#	<0.0001
*C1orf183* *	NM_019099	<0.0001#	<0.0001#	*BUB1B* *	NM_001211	<0.0001#	<0.0001
*CDKN1A* *	NM_078467	0.0007	<0.0001#	*CCNB1* *	NM_031966	<0.0001#	<0.0001#
*FBXO22*	NM_147188	0.0001#	<0.0001#	*CDC25B* *	NM_021873	0.0001#	0.0002
*FBXW7* *	NM_033632	0.0013	<0.0001	*CDCA2*	NM_152562	<0.0001#	<0.0001
*FDXR* *	NM_024417	<0.0001#	<0.0001	*CENPA* *	NM_001809	<0.0001#	<0.0001#
*FHL2*	NM_201555	<0.0001#	0.0003	*CENPE* *	NM_001813	<0.0001#	<0.0001#
*IGFBP4*	NM_001552	0.0003	<0.0001	*FAM65B*	NM_014722	<0.0001#	<0.0001
*MDM2* *	NM_002392	<0.0001#	<0.0001#	*FAM72A*	BC035696	<0.0001#	<0.0001#
*PHLDA3*	NM_012396	<0.0001#	<0.0001#	*FAM83D* *	NM_030919	<0.0001#	<0.0001#
*PLK2*	NM_006622	<0.0001#	<0.0001#	*GTSE1*	NM_016426	<0.0001#	<0.0001
*PLK3* *	NM_004073	0.0007	0.001	*IL16*	NM_172217	<0.0001#	<0.0001
*PPM1D* *	NM_003620	<0.0001#	<0.0001#	*INCENP*	NM_001040694	<0.0001#	<0.0001#
*RGL1*	NM_015149	<0.0001#	<0.0001	*KIF14*	NM_014875	<0.0001#	<0.0001
*SESN1* *	NM_014454	<0.0001#	<0.0001#	*KIF20A*	NM_005733	<0.0001#	<0.0001#
*SESN2* *	NM_031459	<0.0001#	<0.0001#	*KIF23* *	NM_138555	<0.0001#	<0.0001#
*TNC*	NM_002160	0.0334	0.0006	*NEK2*	NM_002497	<0.0001#	<0.0001#
*TNFRSF10D*	NM_003840	0.0172	<0.0001	*PLK1* *	NM_005030	<0.0001#	<0.0001#
*TSGA10*	NM_182911	0.0058	<0.0001#	*PSRC1* *	NM_001032290	<0.0001#	<0.0001#
*VWCE* *	NM_152718	<0.0001#	<0.0001#	*SGOL2*	NM_152524	<0.0001#	<0.0001#
*XPC*	NM_004628	<0.0001#	<0.001#	*SH2D3C*	NM_170600	<0.0001#	<0.001
				*TPX2* *	NM_012112	<0.0001#	<0.001#
				*TROAP* *	NM_005480	<0.0001#	<0.001
				*UBE2C*	NM_181802	<0.0001#	<0.001#

*Genes in common with fibroblasts AS ANOVA 0v10 Gy (Table 4).

# With corresponding adjusted p-values<0.05.

These genes have been called significant in all the three alternative-splicing analysis methods: Partek, FIRMA and Affymetrix's Splicing Index. Exact p-values and adjusted p-values can be found in supplemental materials; only Partek's and FIRMA's p-values are reported.

**Table 4:** Genes predicted to produce alternative transcripts in fibroblast cells 4 hours following 10 Gy of IR.

Up-regulation				Down-regulation			
Gene Sym.	GenBank Acc.	Partek Alt Splice P-Value	FIRMA Alt Splice P-Value	Gene Sym.	GenBank Acc.	Partek Alt Splice P-Value	FIRMA Alt Splice P-Value
*BTG2*	NM_006763	<0.0001#	<0.0001#	*AURKA* *	NM_198433	<0.0001#	0.0002
*C1orf183* *	NM_019099	0.0304	0.0005	*BCOR*	NM_001123385	<0.0001#	0.0185
*CDKN1A* *	NM_078467	<0.0001#	<0.0001#	*BUB1B* *	NM_001211	0.0055	0.0027
*CKAP2*	NM_018204	<0.0001#	0.0112	*C13orf34*	NM_024808	<0.0001#	0.0002
*FBXW7* *	NM_033632	<0.0001#	<0.0001	*CBL*	NM_005188	<0.0001#	0.0068
*FDXR* *	NM_024417	0.0017	0.0001	*CCNB1* *	NM_031966	<0.0001#	0.0002
*GDF15*	NM_004864	<0.0001#	<0.0001	*CCNF*	NM_001761	0.0026	0.0003
*IER5*	NM_016545	0.0001#	<0.0001	*CDC25B* *	NM_021873	<0.0001#	0.0003
*LRDD*	NM_018494	0.0002#	0.0001	*CENPA* *	NM_001809	0.1645	<0.0001
*MDM2* *	NM_002392	<0.0001#	<0.0001#	*CENPE* *	NM_001813	0.0464	0.0005
*PLK3* *	NM_004073	<0.0001#	<0.0001	*FAM83D* *	NM_030919	0.002	<0.0001
*PPM1D* *	NM_003620	0.0011#	<0.0001	*GAS2L3*	NM_174942	0.0002#	<0.0001
*SESN1* *	NM_014454	<0.0001#	<0.0001	*HERC4*	NM_022079	0.0001#	0.0017
*SESN2* *	NM_031459	<0.0001#	0.0002	*HIST1H1T*	NM_005323	0.0307	0.001
*THSD1P*	NR_002816	<0.0001#	0.001	*KIAA1333 (G2E3)*	NM_017769	<0.0001#	0.0267
*TP53INP1*	NM_033285	0.0008#	0.0011	*KIF18A*	NM_031217	0.0248	0.0009
*TRAF4*	NM_004295	0.0004#	0.0005	*KIF23* *	NM_138555	0.0073	0.0084
*VWCE* *	NM_152718	0.004	<0.0001	*PLK1* *	NM_005030	0.0054	0.0003
				*PSRC1* *	NM_001032290	0.0081	0.0006
				*TPX2* *	NM_012112	0.0084	0.0064
				*TROAP* *	NM_005480	0.0288	0.0003

*Genes in common with LCL AS ANOVA 0v10 Gy (Table 3).

# With corresponding adjusted p-values<0.05.

These genes have been called significant in all the three alternative-splicing analysis methods: Partek, FIRMA and Affymetrix's Splicing Index. Exact p-values and adjusted p-values can be found in supplemental materials; only Partek's and FIRMA's p-values are reported.

### Specific gene IR response

**Figure 1:**
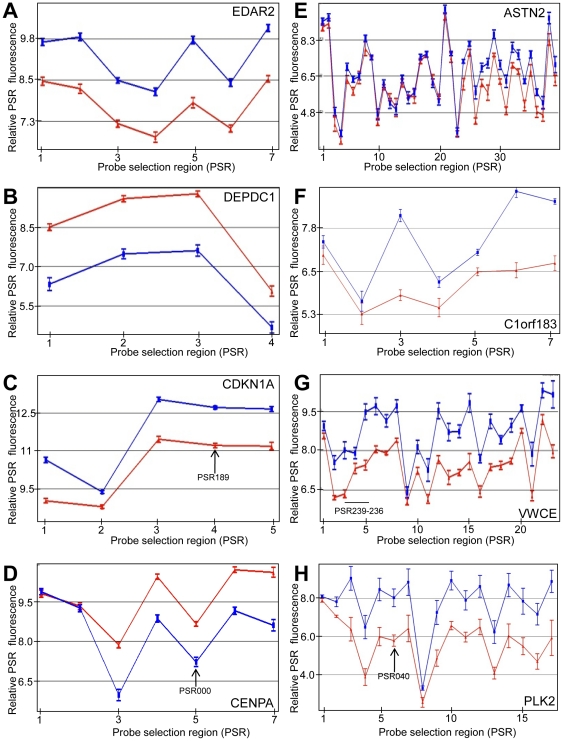
Genes that show modulated transcription expression products, including use of alternative transcripts, after IR in LCLs. Up- (A, C, E–H) and down-regulated (B, D) gene probe selection regions (PSRs) 4 hours following 10 Gy IR in LCLs, which identifies transcript expression at the exon level. Exon expression examples are shown for the following genes: *EDAR2* (A), *DEPDC1* (B), *CDKN1A* (C), *CENPA* (D), *ASTN2* (E), *C1orf183* (F), *VWCE* (G) and *PLK2* (H). Relative PSR flourescence (y-axis) is plotted for each PSR (points along x-axis). Samples were either sham irradiated (red) or irradiated with 10 Gy (blue). PSRs are oriented 5′ to 3′ across the gene from left to right on the x-axis. Relative expression levels are plotted on a log_2_ scale. Arrow represents a PSR or PSR region that was used for subsequent PCR validation. At least 6 cancer patient samples were used for each point (n≥6). Error bars = SEM.

**Figure 2:**
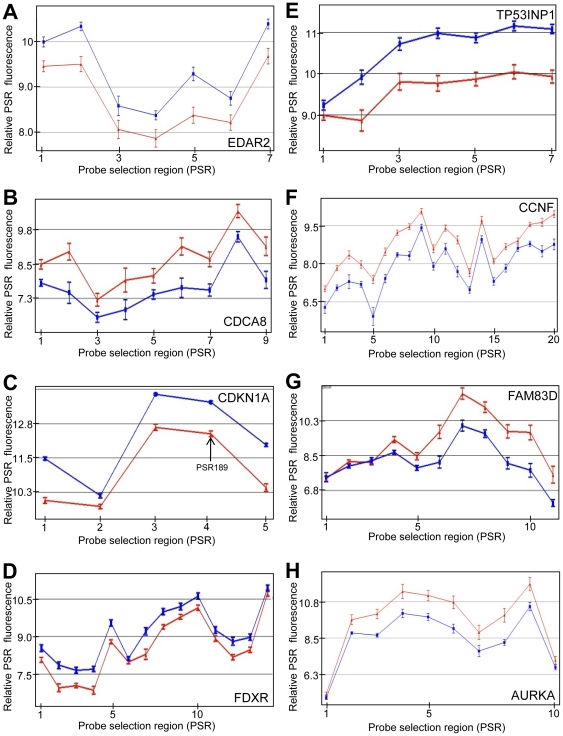
Genes that show modulated transcription expression products, including use of alternative transcripts, after IR in fibroblasts. Up- (A, C–E) and down-regulated (B, F–H) gene probe selection regions (PSRs) 4 hours following 10 Gy IR in fibroblasts, which identifies transcript expression at the exon level. Exon expression examples are shown for the following genes: *EDAR2* (A), *CDCA8* (B), *CDKN1A* (C), *FDXR* (D), *TP53INP1* (E), *CCNF* (F), *FAM830* (G) and *AURKA* (H). Relative PSR flourescence (y-axis) is plotted for each PSR (points along x-axis). Samples were either sham irradiated (red) or irradiated with 10 Gy (blue). PSRs are oriented 5′ to 3′ across the gene from left to right on the x-axis. Relative expression levels are plotted on a log_2_ scale. Arrow represents a PSR or PSR region that was used for subsequent PCR validation. At least 6 cancer patient samples were used for each point (n≥6). Error bars = SEM.

The relative expression of each core PSR for a selection of individual genes with a variety of profiles in LCLs (Figure 1) and fibroblasts (Figure 2) 4 h after 10 Gy IR are shown. In LCLs, *EDA2R* showed a relatively consistent increase at each exon region across the entire gene (Figure 1A), and *DEPDC1* shows a dramatic decrease in transcription across most exon regions (Figure 1B). *CDKN1A* shows an obvious differential increase between exon regions: PSR two is induced less than the rest of the gene and is consistent with a known AS product in *CDKN1A* (Figure 1C). The expression levels of the PSRs in *CENPA* are decreased in response to IR except for the first two PSRs, which show the same expression before and after irradiation (Figure 1D). We observed that the 5′ region of the *ASTN2* gene showed much less induction than the 3′ regions after IR which is consistent with the two main known isoforms for this gene (Figure 1E). *C1orf183* (Figure 1F), *VWCE* (Figure 1G) and *PLK2* (Figure 1H) also have internal PSRs with a differential increase in expression indicating that different isoforms are expressed after IR. These three genes also show the first PSR is not as up-regulated compared to most other PSRs of the transcript (Figure 1F–G).

Analogous investigations using primary fibroblast human cells were performed. A large proportion of genes modulated in the LCLs 4 h after 10 Gy IR were also modulated in the fibroblast cells. *EDA2R*, similar to its expression in LCLs, shows a relatively consistent increase in transcript across the entire gene, although not quite as highly induced (Figure 2A). *CDCA8* is an example of a gene that is down-regulated following radiation (Figure 2B). *CDKN1A* shows a similar pattern as it did in the LCLs (Figure 2C). *FDXR* shows a transcriptional induction across most of the gene with the sixth PSR not showing any induction (Figure 2D). *TP53INP1* shows a large induction except at the first PSR (Figure 2E) which is similar to *C1orf183*, *VWCE* and *PLK2* transcript expression in the LCLs (Figure 1F–H). *CCNF* is generally down-regulated, but some internal PSRs do not show the same degree of decreased expression (Figure 2F). *FAM83D* shows down-regulation after the third PSR and the level of expression decreases more in the PSRs towards the 3′ end of the gene (Figure 2G). *AURKA* also shows down-regulated PSRs relatively evenly throughout the gene with the exception of both the 3′ and 5′ ends (Figure 2H). Therefore, a diverse set of responses are observed in both LCLs and fibroblast cell lines after exposure to IR.

### Validation of the radiation response using PCR

**Figure 3:**
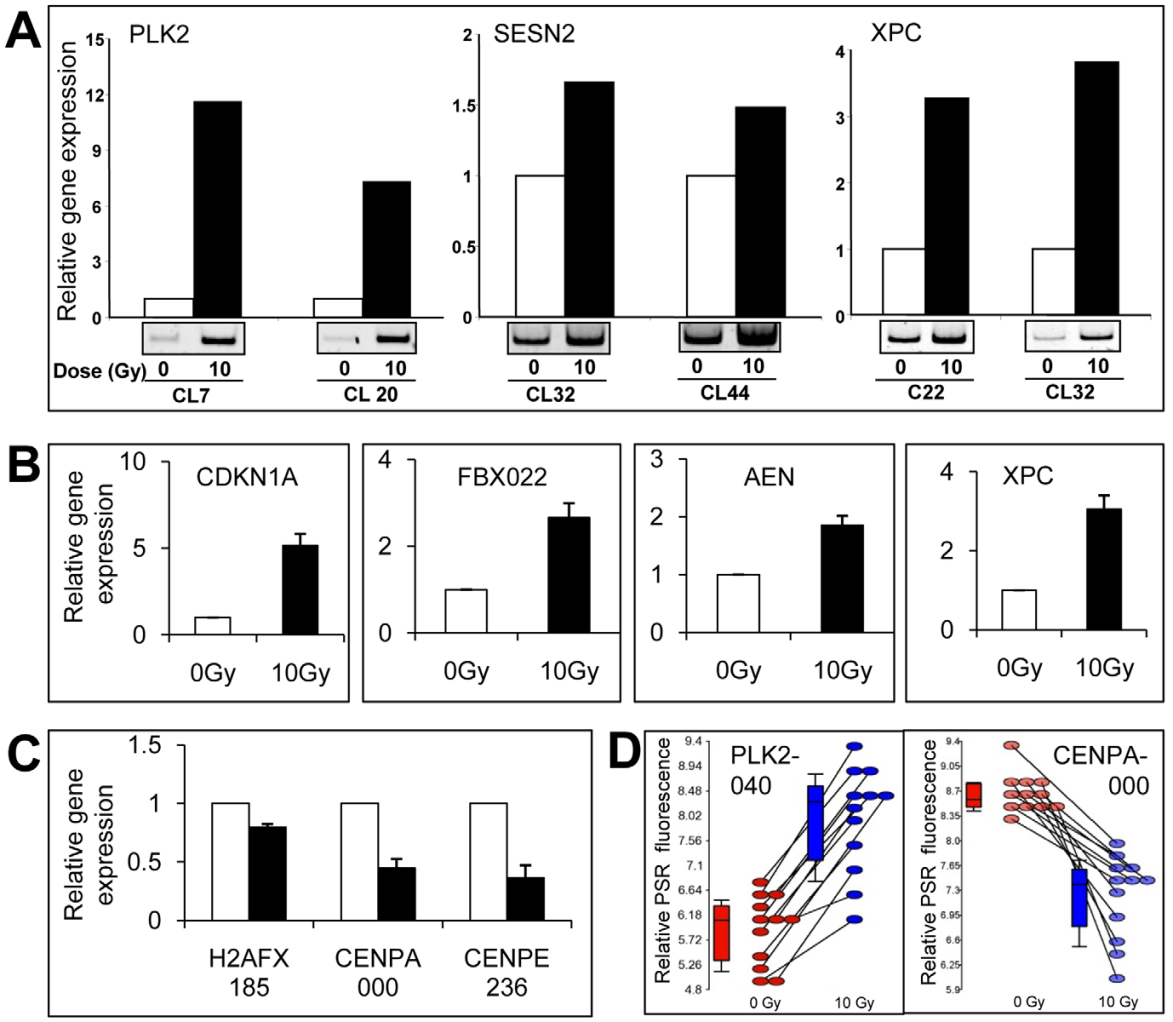
PCR validation of ionizing radiation responsive genes in LCLs. (A) PCR was used to amplify the *PLK2*, *SESN2* and *XPC* cDNA derived from the transcriptional products of cell lines that were irradiated with 10 Gy or sham irradiated. The amplified products were analysed by polyacrylamide gel electrophoresis. The relative amounts were calculated using densitometric analysis and expression levels were normalized to PGK expression. QRT-PCR was used to validate selected up- (B) and down- (C) regulated genes. PSRs that were used to assess intra-gene expression are indicated by the last three numerals of the gene-specific PSR. Error bars represent the SEM. Bar graphs represent *CDKN1A*-PSR189: n = 4 (p = 0.012); *FBXO22*-PSR527: n = 6 (p = 0.004); *AEN*-PSR256 (p = 0.003); *XPC*-PSR853 (p = 0.0001); *H2AFX*-PSR185: n = 6 (p = 0.001); *CENPA*-PSR000: n = 5 (p = 0.002); *CENPE*-PSR236: n = 4 (p = 0.002). (D) Example of individual cell lines that show increased or decreased expression at a specific PSR following radiation are shown. *PLK2*-PSR040 (induced) and *CENPA*-PSR000 (down-regulated) array data expression levels for each LCL tested at a representative PSR are shown at 0 (red) and 10 (blue) Gy. Lines link 0 Gy and 10 Gy for the individual cell lines. Boxes in box plots show 50% and whiskers to 80% of samples.

**Figure 4:**
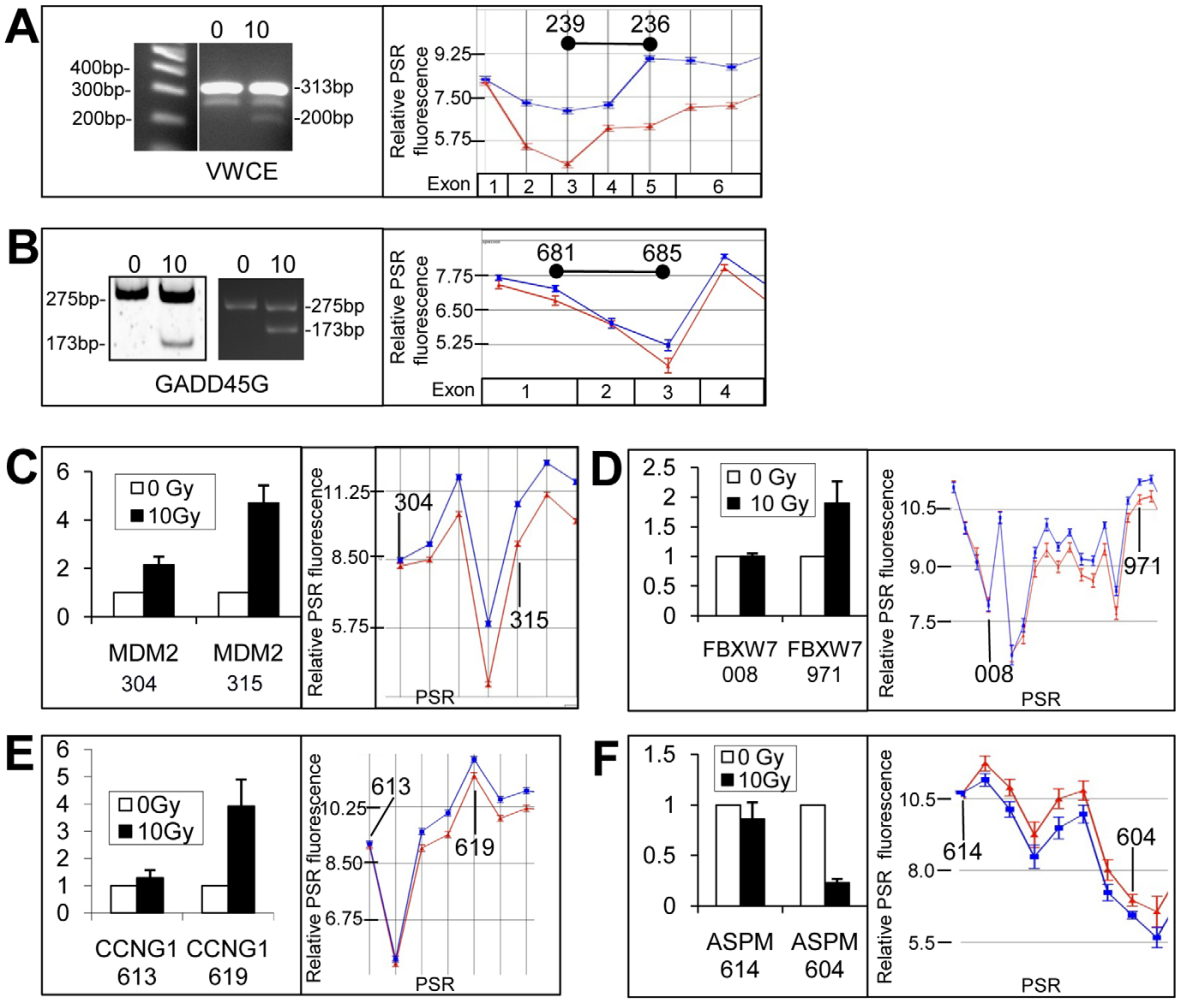
Alternative transcripts identified in LCLs using exon microarrays were verified using PCR techniques. Primers were designed to span across exons of genes (*VWCE*: PSR239-236; *GADD45G*: PSR681-685) to yield alternative amplicons for AS as indicated by microarray AS graphs (A and B). Gene expression graphs for these genes are shown to the right with amplicons indicated (black bars). The corresponding exons of the gene are indicated in the boxes below. QRT-PCR was used to amplify specific PSRs to validate expression differences for up- (C–E) and down- (F) regulated intra-gene transcript expression differences. PSRs that were used to assess intra-gene expression are indicated by the last three numerals of the PSR following the gene symbol. Graphed microarray expression data is shown for sham treated samples (red lines) or samples isolated 4 hours post 10 Gy IR (blue lines). Only partial regions of genes are shown in expression line graphs. Error bars represent the SEM and n = 12 for each sample in the line graphs. Bar graphs sample numbers are as follows: *MDM2*, *FBXW7* and *ASPM*: n = 6, and for *CCNG1*: n = 3. Relative gene expression values are plotted on the y-axis for panels A–D bar graphs. Error bars in bar graphs represent the SEM.

**Figure 5:**
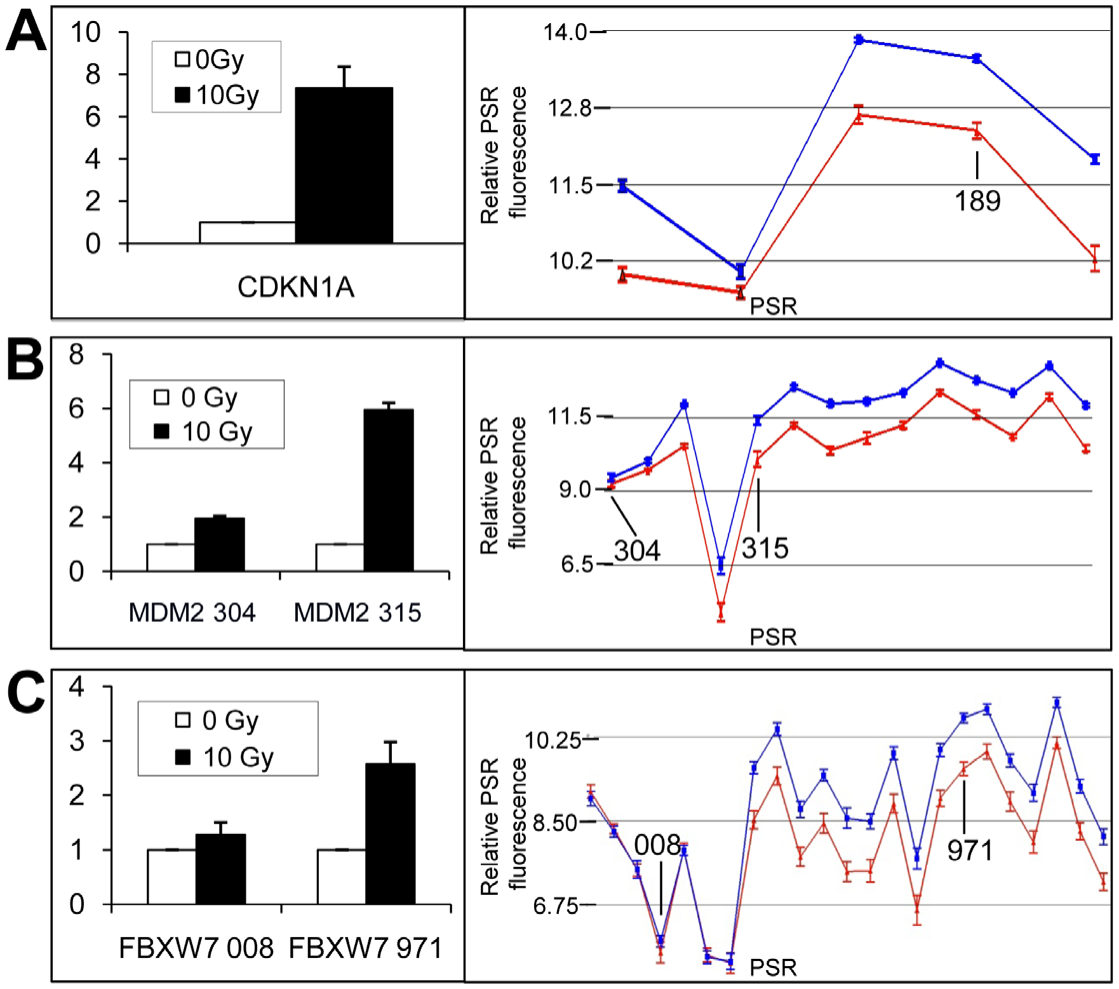
PCR validation of ionizing radiation responsive genes in human primary fibroblasts. QRT-PCR was used to amplify specific PSRs (as indicated on expression line graphs) to validate expression differences in specific PSRs (A–C) including intra-gene transcript expression differences (B and C). Error bars represent SEM. *CDKN1A*: n = 9 (p-value = 0.0002); *MDM2*: n = 10 (p-value of induction = 0.0004; p-value for difference between PSRs = 0.0033); and *FBXW7*: n = 12 (p-value of induction = 0.0038; p-value for difference between PSRs = 0.0004). PSRs that were used to assess intra-gene expression are indicated by the last three numerals of the PSR following the gene symbol. Graphed microarray expression data is shown for untreated samples (red lines) or samples isolated 4 hours post 10 Gy IR (blue lines). PSRs used for intra-gene expression difference validation are shown in these graphs. Relative gene expression values are plotted on the y-axis for panels A–C bar graphs.

Expression differences observed from microarrays at 4 hours after exposure to 10 Gy of radiation was validated by PCR using several different LCLs (3 to 12 for QRT-PCR) as indicated (Figures 3, 4, 5). Primers were designed within exon regions (Table S1). QRT-PCR primers to *PGK* and/or *GAPDH* transcripts were used for normalization controls. Amplicons from genes (e.g., *PLK2*, *SESN2* and *XPC*) were run on polyacrylamide gels using cycle numbers determined to be in the linear amplification range (Figure 3A). Primers were prepared to selected PSRs and QRT-PCR was performed to compare transcript levels in sham-irradiated and 10 Gy at 4 hours post-IR. The results for a number of gene transcripts shown to be modulated from exon array data were confirmed to be induced (Figure 3B) or down-regulated (Figure 3C). Likewise, similar validation experiments were conducted to confirm microarray data obtained for fibroblast samples (Figure 5). Examples of relative expression levels for each of twelve cell lines for sham-irradiated and 4 hr post-IR at a specific PSR is shown for *PLK2* PSR040 and *CENPA* PSR000 (Figure 3D).

### Validation of AS using PCR

Selected genes shown by exon array AS algorithms to have altered transcripts following radiation were verified using PCR methods. *VWCE* gene exon array results suggested this gene was alternatively spliced. A region from PSR235 to PSR239 was amplified with PCR and the resulting amplification products run on an agarose gel. A 313 bp band full length product, as well as a 200 bp shorter product found only in the 10 Gy sample was observed (Figure 4A). Bands were extracted from the gels and sequenced. The bands were found to be the predicted full length and an alternatively spliced transcript, missing exon 4. Similar banding was obtained in three separate patient samples. Likewise, the *GADD45G* amplicon spanning PSR681 to PSR685 was amplified with PCR and amplicons were run on a polyacrylamide gel. Both the 0 and 10 Gy samples for two separate LCLs showed bands at 275 bps and the 10 Gy sample had an additional shorter band at 173 bps (Figure 4B). Again, sequencing confirmed that the lower product was an alternatively spliced form that was missing exon 2. Identical banding patterns were observed in six separate patient samples. Both these examples showed in frame exon skipping.

QRT-PCR was also used to validate exon array predicted alternative transcripts induced following radiation. PSR expression levels for *MDM2* (Figures 4C and 5B), FBXW7 (Figures 4D and 5C) and *CCNG1* (Figure 4E) were consistent with alternative spliced products as predicted from the exon expression arrays. 5′-RLM-RACE, which only amplifies capped mRNA was performed for the *MDM2* transcripts. We found the predicted size amplicon for alternative start site use and confirmed this with sequencing. Some genes such as *ASPM* were down-regulated in response to IR, however, often we observed that some specific PSRs were not down-regulated. For example, compare *ASPM*-PSR614 to *ASPM*-PSR604; expression levels were analysed using QRT-PCR to verify these findings (Figure 4F).

Additional methods for identifying alternative spliced products included FIRMA and SI tests (Tables S6, S7, S8, S9, S10, S11). These methods revealed a high correlation with the Partek Genomics Suite AS algorithm. Genes that were identified to have IR-induced alternative transcription, using all three analysis methods, included the up-regulated genes, CDKN1A, *IER5*, *MDM2*, *PLK2*, *SESN1* and *SESN2* (Tables 3 and 4; Figures 1, 2, 4 and 5). We also identified down-regulated genes with differential PSR expression (e.g., *ASPM* (Figure 4F), *CCNB1*, *CCNF*, *CENPA*, and *PLK1*; Tables 3 and 4; Figures 1 and 2).

### LCL vs Fibroblasts

**Figure 6:**
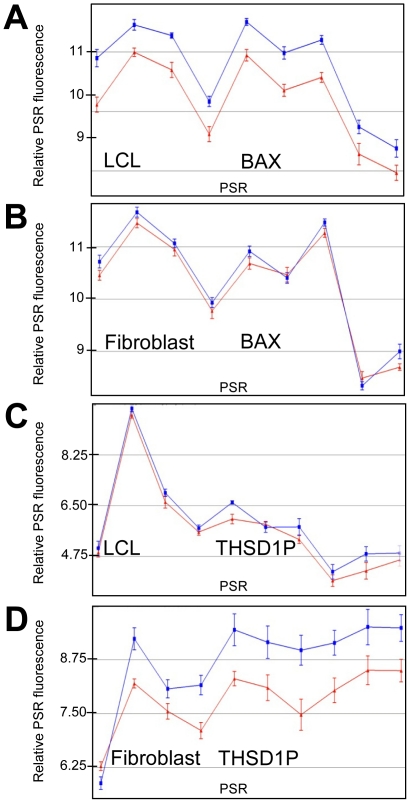
Lymphoblastoid and fibroblast cells show different transcription responses to IR. Gene expression (as determined from exon microarrays) across the *BAX* (A, B) and *THSD1P* (C, D) genes are graphed for each PSR 4 hours after 10 Gy IR (blue line) or sham treated (red line) in LCLs (A, C) or fibroblasts (B, D). n = 6 and SEM is graphed for each PSR.

There is a high degree of overlap for radiation modulated whole gene expression between LCLs and fibroblasts. *ED2A* (Figures 1A and 2A), *CDKN1A* (Figures 1C and 2C), *MDM2* (Figures 4C and 5B) and *FBXW7* (Figures 4D and 5C) are a few examples. However, there are genes that show cell type specific modulation in response to IR 4 hours after exposure. For example, *BAX*, *BCL2*, *RRM2B* and *ATF3* are induced in LCLs but not in fibroblasts, and *THSP1* and *PAG1* are induced in fibroblasts but not LCLs. Two representative genes (*BAX* and *THSD1P*) are shown in Figure 6. Also, there is generally a more robust expression response at four hours post-IR across the whole gene in LCLs compared to fibroblasts. For example, the fold change for the top 20 up-regulated genes 4 hours after 10 Gy IR range from 1.72 (for *TNFRSF10B*) to 3.79 (for *PLK2*) for LCLs (Table 1) and only from 1.36 (*ZNF79*) to 2.63 (*CDKN1A*) in fibroblast cells (Table 2). Similarly, the fold change for the top down-regulated genes 4 hours after 10 Gy IR ranged from −1.38 (*INCENP*) to −4.63 (*KIF20A*) for LCLs (Table 3) and only −1.19 (*SETD8*) to −2.63 (*AURKA*) for fibroblasts (Table 4). Also the difference in the robustness of the results between LCL and fibroblasts is apparent when comparing the values of fold change between some genes common to both lists. For example, *CDKN1A* which has a fold increase of 2.94 in LCLs compared to 2.63 in fibroblasts, and *EDA2R* has a fold change of 2.67 in LCLs compared to 1.57 in fibroblast cells (Tables 1 and 2).

### IR dose response

**Figure 7:**
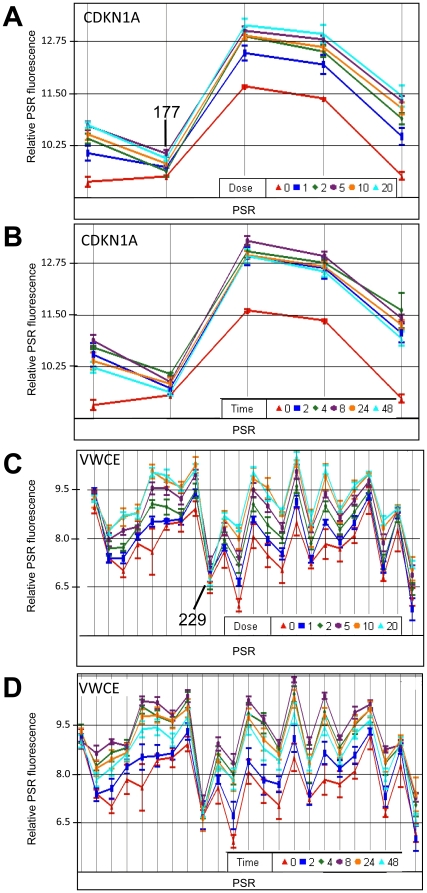
Time course and dose response of gene transcripts induced in LCLs by IR as determined from exon level microarrays. Transcripts for *CDKN1A* and *VWCE* were isolated 4 hrs after exposure to 1, 2, 5, 10 or 20 Gy (A, B) of ionizing radiation or exposed to 10 Gy IR and transcripts isolated 1, 2, 4, 8, 24 or 48 hours post-IR (C, D). Relative expression (y-axis) is plotted for each PSR (points along x-axis). PSRs are oriented 5′ to 3′ across the gene from left to right. Relative expression levels are plotted on a log_2_ scale. 12 cancer patient samples were used for each point (n = 12). Error bars = SEM.

**Figure 8:**
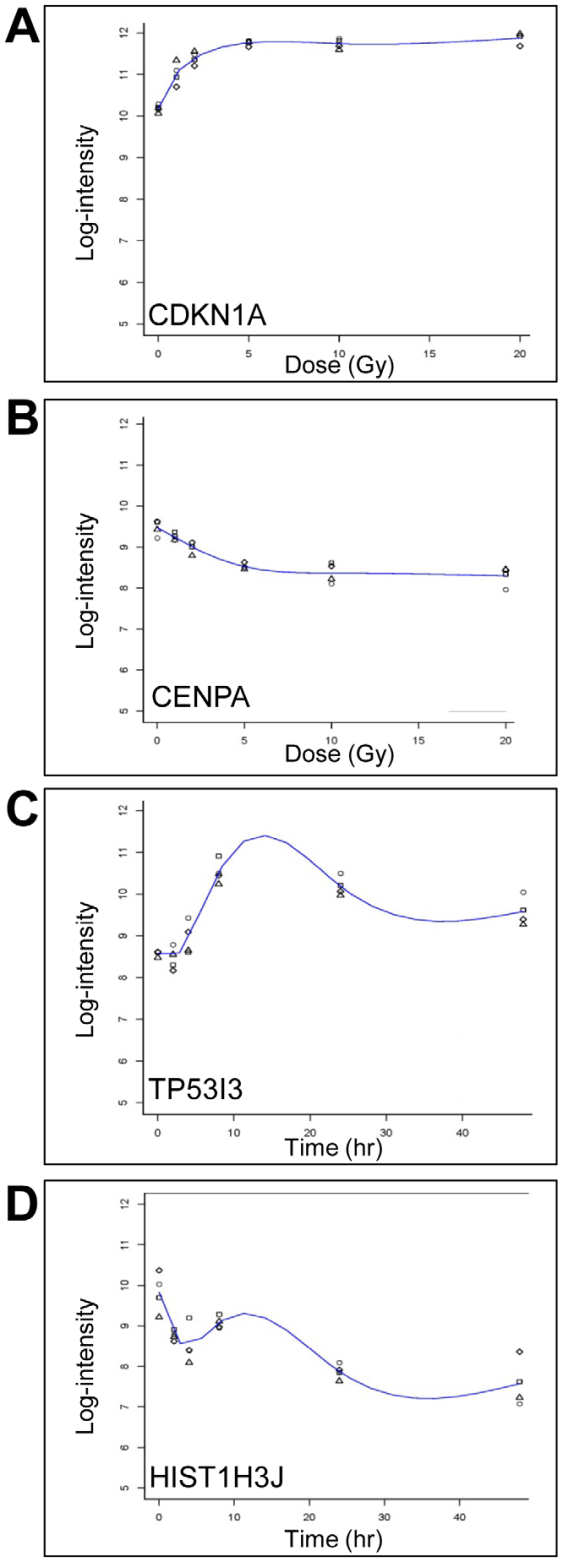
Dose response and time course graphs of whole gene expression. Examples of gene expression as a function of log2-intensity of fluorescence is plotted for *CDKN1A* and *CENPA* transcripts from LCLs isolated 4 hrs after exposure to 0, 1, 2, 5, 10 or 20 Gy (A, B) of ionizing radiation or exposed to 10 Gy IR and *TP53I3* and *HIST1H3J* transcripts isolated 0, 1, 2, 4, 8, 24 or 48 hours post-IR (C, D). Relative expression as determined from exon microarrays (y-axis) is plotted for each dose and time point. Each dose or time point has four samples shown and a line was fit to these data points.

Investigation of dose response was completed using a range from 1 Gy to 20 Gy of IR. RNA was collected at 4 hours post-IR, processed and run on exon arrays for four cell lines for each cell type. Examples of genes that showed modulation with dose are plotted (Figure 7A and 7C). In general, the responses increased with dose, however, it was common for a gene to show substantial modulation at the low dose (1 Gy) with less relative modulation at increasing doses. For example, in LCLs, *CDKN1A* showed a strong induction at 4 hours after 1 Gy and then gradually increased with increase in radiation dose (Figure 7A). Also, the alternatively spliced form was clearly present at all doses. For example, compare *CDKN1A*-PSR177 to the other PSR expression level changes (Figure 7A). The *VWCE* transcript showed induction in response to radiation at every dose, but the induction was more gradual compared to *CDKN1A* (Figure 7C). *VWCE*-PSR229, a region that did not change much in response to IR, was modulated similarly at all doses. Whole gene expression with varying dose was also determined. Selected genes are plotted that show a variety of kinetics for gene dose responses (Figure 8A and 8B; Tables S12 and S13).

### Time course

The effect that time had on the transcript levels was also investigated using time points spanning from 2 to 48 hours post-IR (10 Gy) in four cell lines for both LCL and fibroblast samples. Selected genes, that show a modulation with time, are plotted (Figure 7B and 7D). Exon arrays revealed that genes such as *CDKN1A* showed a very early and robust response to radiation which was maintained over the whole time course up to two days. The alternative spliced form was also evident at all times tested (Figure 7B). Unlike *CDKN1A*, *VWCE* showed a more linear increase with time up to 8 hours and then was observed to drop down again. The alternatively spliced form was evident for all times (Figure 7D). Whole gene expression with varying time points was also determined. Selected genes are plotted that show different types of time course transcription responses (Figure 8C and 8D; Tables S14, S15, S16, S17). Many genes from the gene families HIST, MCM and E2F, were found to be down-regulated in response to IR. Many of the histone genes (e.g., *HIST1H3H*, *HIST1H3F*, *HIST1H2BM, HIST1H4F*, *HIST1H2BB*, *HIST1H3J* and *HIST1H2AB*) have not previously been reported to be modulated by IR (Figure 8D; Table S4).

### IR modulation of genes at the chromosomal level

**Figure 9:**
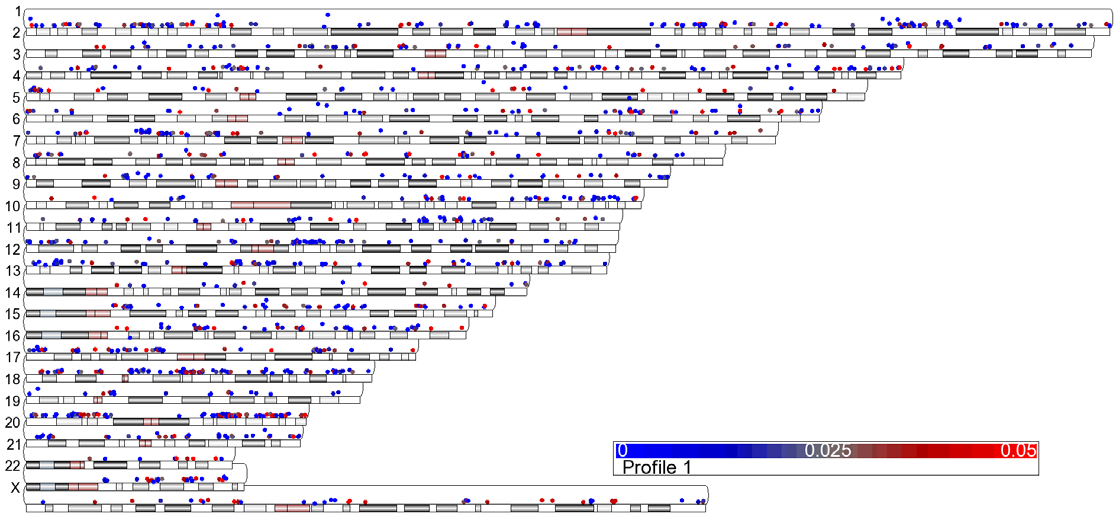
Chromosome location of genes modulated by IR in LCLs. 12 LCLs were irradiated with 10 Gy or sham IR and RNA was isolated 4 hours post-IR. Genes with significant (p-value (Dose)<0.05) up-regulated (blue circles) and down-regulated (red circles) 4 hours after 10 Gy IR are plotted above the chromosome location. Chromosome number is indicated on the right of the diagram.

**Figure 10:**
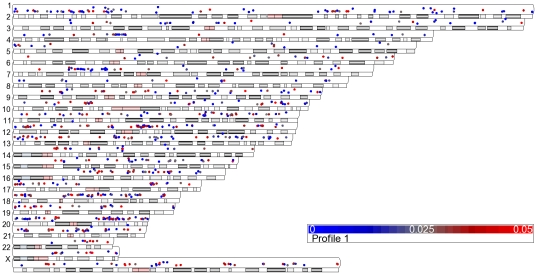
Chromosome location of genes modulated by IR in fibroblast cells. 12 fibroblast cells were irradiated with 10 Gy or sham IR and RNA was isolated 4 hours post-IR. Genes with significant (p-value (Dose)<0.05) up-regulated (blue circles)and down-regulated (red circles) 4 hours after 10 Gy IR are plotted above the chromosome location. Chromosome number is indicated on the right of the diagram.

**Figure 11:**
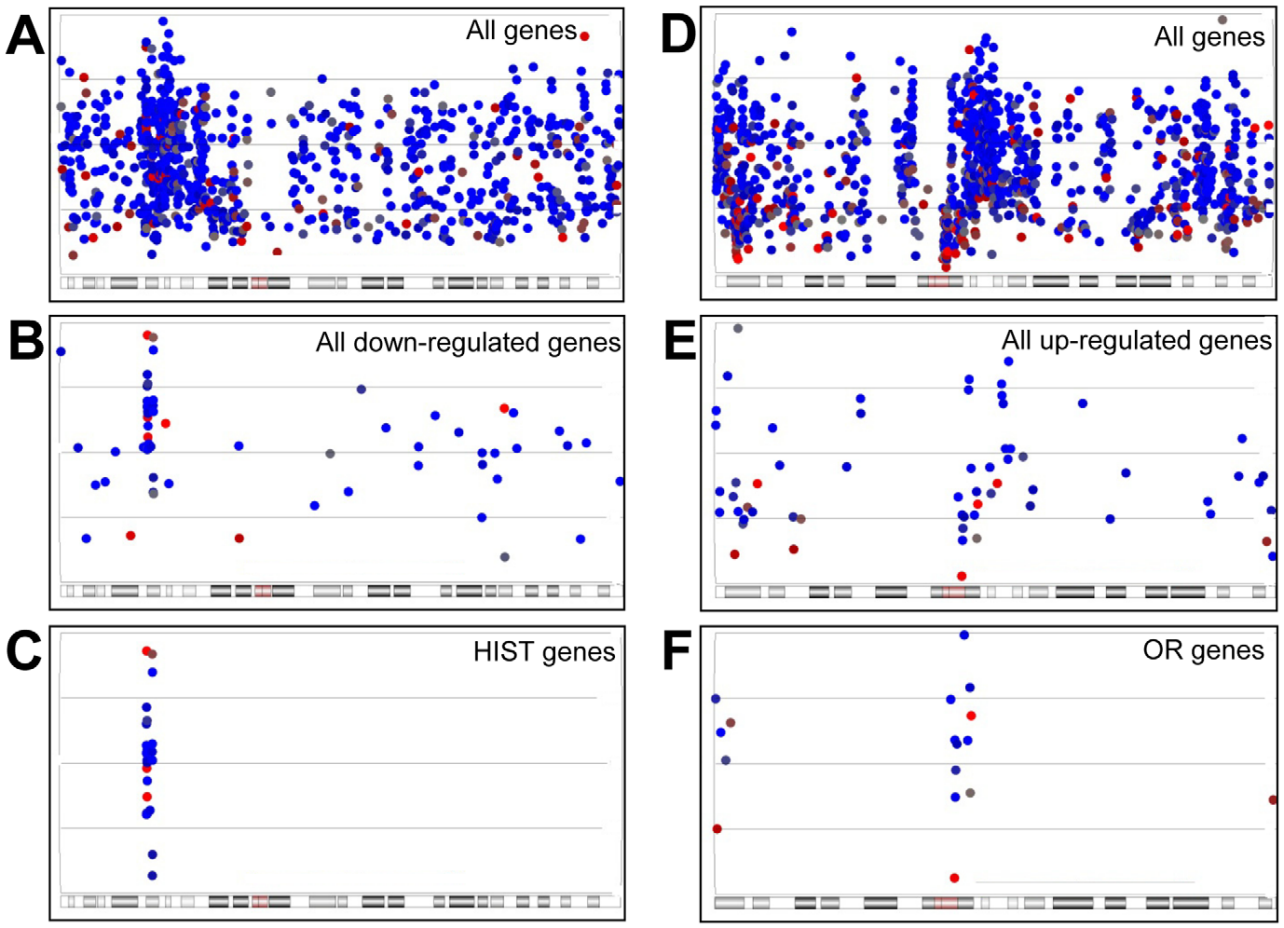
Chromosomal location of clustered radiation responsive genes. All genes represented on the exon array that are found on chromosome 6 are shown (A). Four hours after 10 Gy IR, down-regulated genes in LCLs were selected based first on p-value of <0.1 and then the top 500 genes based on fold change were selected and plotted (B). Only the HIST genes (n = 21) on chromosome 6 that are down-regulated in LCLs are plotted (C). Likewise, all genes represented on the exon array that are found on chromosome 11 are shown (D). Four hours after 10 Gy IR, up-regulated genes were selected based first on p-value of <0.1 and then the top 500 genes based on fold change were selected and plotted (E). Only the olfactory receptor genes (OR; n = 17) on chromosome 11 that are up-regulation in LCLs are plotted (F). Blue and red filled circles represent individual genes which made the selection. Colour is based on p-value, blue indicating a lower p-value than red. Chromosome cytobands are represented below the plots with the p-arm of the chromosome towards the right and the q-arm towards the left of the diagram.

The location of IR-modulated genes for each chromosome was determined to identify regions that may have IR-specific regulation. In general, responsive genes four hours post-IR were present throughout the chromosomes and more so in gene-rich regions. Some chromosomes had regional clusters of radiation responsive genes. For example, chromosomes 6 and 11 have regions that show enriched gene expression modulation after IR (Figures 9 and 10). Chromosome 6 has a region enriched for IR-modulated genes in LCLs, many of which are down-regulated HIST genes (Figure 11A–C). In this gene rich region there are locations just adjacent to the HIST cluster for which relatively few genes are down-regulated even though there are many more genes present than *HIST* genes in the HIST cluster. Of the 61 genes down-regulated on chromosome 6 (p-value<0.1 and 500 top based on fold change), we found 21 (38%) HIST genes, all of which were found at the HIST cluster on chromosome 6. We found down-regulation of HIST genes in both LCL and fibroblast cells although to a lesser extent in the fibroblasts. The gene olfactory receptor gene clusters on chromosome 11 showed many of these genes to be up-regulated (17/61 (28%) up-regulated genes (p-value<0.1 and 500 top based on fold change)) (Figure 11D–F). Comparison of the expected frequencies to the actual genes modulated after IR varied between chromosomes and cell lines. For example, a lower than expected number of radiation responsive genes were found in chromosomes 4, 13 and 21 in LCLs and a particularly higher number than expected were observed for chromosome 18 in fibroblasts (Table S18). There was an overall variation between cell types and between individual chromosomes such as chromosomes 13 and 18 (Table S18).

### Gene ontologies and gene networks

**Figure 12:**
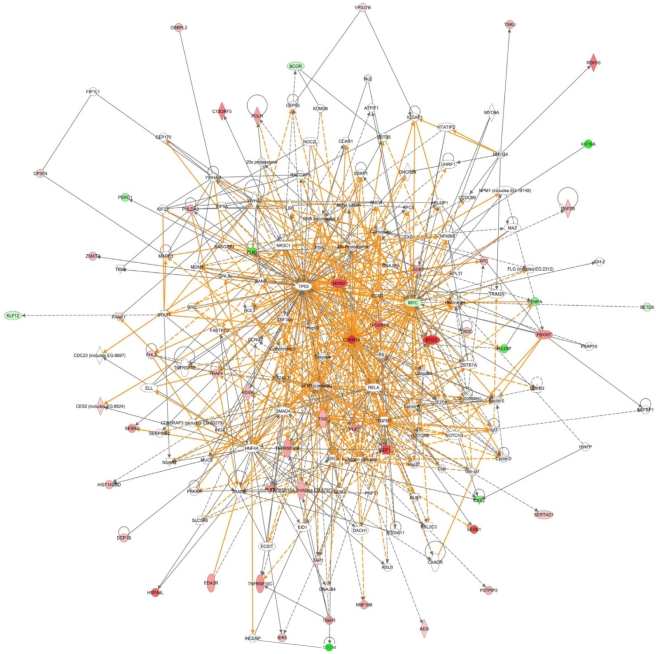
Gene network in response to IR. Genes such as *CDKN1A* and *MDM2* are central in this network. Solid lines represent direct protein interactions and dashed lines represent indirect interactions. Different shapes correspond to different gene ontological groupings (Ingenuity Pathway Analysis). Shaded genes are genes that are in the top 100 up-regulated (red) and top 100 down-regulated (green) genes shown to be modulated at 4 hours following 10 Gy IR in LCLs.

Gene functional ontologies for the IR regulated genes were determined and cell cycle, cellular assembly and organization, DNA replication, recombination and repair, cell death and cellular movement were the top 5 functional categories in LCLs as determined by Ingenuity Pathway Analysis (Figure S1A). p53 signalling, molecular mechanisms of cancer, and cell cycle: G2/M DNA damage checkpoint regulation were the top three pathway categories (Figure S1B). Gene functional ontologies for the IR regulated genes were also determined in the fibroblast cells and the top categories were similar to the LCL cells, with cell death as the most prominent, and cell morphology also ranked highly (Figure S1C). p53 signalling also was the top pathway for the fibroblast IR response gene set (Figure S1D). Networks of the top 100 up- and down-regulated genes were determined with the IPA package. A large network, revolving around p53 (although p53 itself was not modulated significantly at the transcript level), *CDKN1A*, cyclins, TNF and PLK genes, was obtained (Figures 12 and S2).

## Discussion

### Whole gene transcript modulation following IR

Gene transcription at the exon level in response to IR has been determined enabling identification of alternative transcription products across the whole genome in response to IR. Genes have been identified that show expression differences between exons in response to radiation. These results are consistent with previously reported alternative transcript gene products in response to IR for single gene examples [35], [36], [37], [41]. Furthermore, analysis of these IR-modulated gene responses across dose and time revealed specific patterns of expression. These responses, in some cases appeared to be a general feature of responses to IR, as revealed by the similar pattern of expression of many genes between cell types, but also cell type specific responses were identified. Furthermore, expression at the chromosomal level revealed certain chromosomal regions that are enriched for radiation responsive genes. The comprehensive probe coverage led to a highly sensitive microarray and also enabled the identification of genes not previously reported to be significantly modulated by IR. Six of the down-regulated genes in fibroblast cells (*BCOR*, *CBL*, *FAM100B*, *FAM72A*, *SETD8* and *TIGD1*) have low fold changes between −1.19 and −1.64 which may have made them difficult to identify as significantly modulated genes in previous studies. Similarly the five up-regulated genes in FB (*ASAH3L* (*ACER1*), *EDA2R*, *PAG1*, *RNF19B* and *XPC*) were among the genes with smaller expression differences on our list ranging from a fold increase of 1.39 to 1.57. However, the fold changes for the novel genes in LCLs are not as low. The differences in cellular responses, namely that the LCLs are more radiosensitive than fibroblasts, are at least in part due to the different gene expression response elicited by these two cell types.

### Alternative transcripts induced by radiation

We have identified a large number of genes that show transcript expression modulation characteristic of alternative transcripts in response to IR. Some genes that show AS following IR have important functional roles in cell fate decisions in response to IR-induced cell and DNA damage. Our array data indicate that genes such as *VWCE* and *GADD45G* produce increased amounts of alternative transcripts lacking internal exons in response to radiation. These proteins are involved in signalling pathways, *GADD45G* being involved in the p38/JNK pathway. Our array data analysis suggests an IR specific induction of alternative transcripts for many genes. The genes identified include involvement in cell cycle regulation, chromatin dynamics, p53 regulation and cell growth. We have identified other genes that show complex transcript isoforms prior to and in response to IR. Exon arrays have the limitation that one cannot know the isoforms when multiple isoforms are present without thorough investigation using other methodologies such as PCR. The array data has given important clues for which exons are involved in AS. For genes, such as *CDKN1A*, *SUN2*, *LRDD* and *SAT2*, we were able to identify alternatively spliced products, and we showed an induction of both products following radiation, but failed to show a difference in the ratio of isoforms after IR. This may be limited by the sensitivity of our PCR assay (not quantitative PCR) or these differences may be teased out at different times or doses. Nevertheless, an induction of an alternative isoform can also be an important contributor to the cellular response to radiation.

Some genes show patterns consistent with induction of alternative start sites such as *VWCE*, *FBXW7*, *CCNG1* and *MDM2* (Figure 2A, C–E). The use of an alternative promoter has been observed for the *MDM2* gene [35], and these investigations strongly support the idea that alternative start site and probably alternative promoter usage is a common feature of transcript regulation in response to IR which certainly has functional downstream consequences for many proteins, either by altering functional groups, initiation of translation and/or RNA stability. Many of the genes that show this have p53 binding elements at or near the induced start site. We propose that the use of an alternative promoter to produce isoforms is a general strategy used by the cell in response to various stress events or changes in the cell environment for which these types of alternative transcripts can affect cell fate or action. p53 is likely to contribute to alternative transcription start sites as has been shown for the *MDM2* gene. The genes that make use of alternative transcription start sites are a different set of genes from those that show AS due to RNA polymerase II slow-down [25]. The genes with IR-induced alternative transcription start sites identified here are genes that are up-regulated, and therefore may have a more active role in regulating the cellular response to genotoxic stress.

### Down-regulated transcript regions

We observed that many transcripts are down-regulated in response to IR. This is evident in many genes involved in the cell cycle and undoubtedly reflects, at least in part, the well known cell cycle delays induced by IR. A feature identified in this investigation by probing at the exon level is that for some regions of transcripts that are, in general, down-regulated, often show just one or part of an exon, without an expression decrease after IR exposure. This was commonly observed in the first exon or two as is evident for *CENPA* (Figure 1D) and *ASPM* (Figure 4F) in LCLs and *FAM83D* (Figure 2G) in fibroblasts, and both the 5′ and 3′ end of *AURKA* in fibroblasts (Figure 2H). It is possible that these regions, which are often at the first exon, are protected from degradation by the transcription machinery or other DNA binding factors and associated proteins. This would be consistent with RNA polymerase II slow-down for some genes [25]. Alternatively, RNA secondary or tertiary structures may prevent degradation. It is possible that the presumed short RNA transcripts may act in a regulatory manner analogous to inhibitory RNAs. Chromatin state may also play a role. Other gene groups were also found to be co-ordinately down-regulated in response to IR. These include histone genes which are regulated by NPAT during a replication block [42], [43] and MCM genes involved in DNA synthesis [44].

### LCL vs Fibroblasts

LCLs and fibroblasts respond differently to the same radiation treatment, and therefore, have a cell type specific response to IR. We found that many transcripts had an exaggerated response in LCLs compared to fibroblasts and some transcript responses differed dramatically between the two cell types (e.g., *BAX*, *THSD1P*, *RRM2B*, *PAG1* and *ATF3*). Some of these response differences may contribute to the higher cellular radiosensitivity that LCLs have compared to fibroblasts. Consistent with this, genes involved with apoptosis were induced more in LCLs compared with fibroblasts. Alternatively, these two cell types may have different thresholds for advancing certain fate decisions such as cell death. For instance, the more robust induction of apoptosis genes in LCLs compared to the fibroblasts, may be the key to higher LCL radiosensitivity.

### Dose response and time course

A variety of expression kinetic profiles were observed with dose and time course studies. For example, a number of genes (e.g., *CDKN1A*; Figure 7A) showed an increase in response with increasing doses until a plateau was reached at the higher doses. Some down-regulated genes showed an analogous down-regulated profile (e.g., *CENPA*). Many genes that had relatively large expression change tended to peak at around 8 hours, for example, *TP53I3*. Finally, groups of related genes were co-ordinately down-regulated as is the case for HIST genes. Therefore, there are many patterns of co-ordinately regulated genes which direct the cellular response to IR.

### IR modulation of genes at the chromosomal level

We also investigated the distribution of IR-modulated genes across the genome and found that some chromosomes had relatively high levels of IR responsive genes. We also found some chromosomal regions show enriched regions of IR-modulated genes. This may indicate that there are genes that are co-ordinately regulated by changes in chromatin structure. Possible mechanisms include changes in methylation or acetylation levels, or activation of other factors that can affect chromatin accessibility to transcription factors. Also, known gene clusters were identified, for example, the down-regulated histone cluster on chromosome 6, and the up-regulated olfactory genes on chromosome 11. Histone changes are likely to be due to changes in DNA synthesis [45]. The reason for the coordinated regulation of olfactory genes is unknown but could be due to chromatin modification. These types of responses at the chromatin level enable large numbers of genes to be turned on and off co-ordinately for major responses to IR such as cell cycle/replication blocks.

### Gene ontologies

We have shown that many of the cell cycle regulatory genes are modulated in response to radiation including many that suggest increased alternative transcript isoform production after irradiation. Modulation of cell cycle genes was prominent in both cell types and is not unexpected since radiation is known to induce a cell cycle block to allow for DNA repair. Cell death is another functional category which is represented in both cell types. Cell death is one mechanism the cell utilizes to eliminate cells that have too much damage.

### Gene networks

The response to radiation is very robust and rapid. With this comprehensive data set we have been able to generate a transcription network of genes modulated by IR in two different cell types. We found that many of the genes that were modulated in response to radiation are linked to the p53-mediated pathway. Consistent with previous observations, *PLK2* and *PLK3* were both robustly up-regulated [6], [46] whereas *PLK1* was down-regulated in response to IR [47]. Other p53 responsive genes up-regulated include: *ATF3*, *BTG2*, *CDKN1A*, *GADD45A*, *MDM2*, *RRM2B*, *SESN1*, *SESN2*, *TP53INP1* and *TP53I3*. Many genes from the TNF family also showed modulated expression levels in response to IR.

The use of alternative transcription start sites may be a global mechanism using alternative promoters to increase the level of certain proteins and protein isoforms required for the radiation response. Other alternative transcript mechansims, such as alternative splicing products in response to IR is an additional way to regulate appropriate cellular action. These studies have also elucidated other novel features of the radiation response such as potential RNA fragment protection and chromatin regulatory roles. Furthermore, these novel aspects of the response to IR may be applicable to other DNA damaging agents and cell stressors in general.

## Materials and Methods

### Cell lines

Epstein-Barr virus transformed lymphocytes were made from lymphocytes derived from cancer patient blood as described [48], [49], [50], [51]. Primary fibroblast cells were derived from human skin biopsies as previously described [48]. LCLs were grown in RPMI medium and fibroblasts in DMEM medium, both supplemented with 10% FBS and gentamicin and incubated in a 5% CO_2_ humidified incubator. 1×10^8^ cells were irradiated with 0, 1 Gy, 2 Gy, 5 Gy, 10 Gy or 20 Gy and RNA was isolated at 0, 2, 4, 8, 24 or 48 hours post-IR. A Cs^137^ source with a dose rate of 1.7 min/Gy was utilized to irradiate the cells at room temperature. Cells were in log phase growth and fibroblasts were about 80% confluent when irradiated. All patients have given written informed consent and studies have been approved by the Peter MacCallum Cancer Centre and Monash University Ethics Committees.

### RNA Isolation

Ten million cells were pelleted, resuspended in 3 ml PBS and an equal volume of Trizol (Invitrogen, Carlsbad, CA, USA) was added, mixed and the aqueous layer was mixed with and equal volume of 70 percent ethanol and added onto a RNeasy column (Qiagen, Venlo, The Netherlands). The RNA extraction was continued by using the RNeasy method as per manufactures recommendation except starting with the addition of the sample of Buffer RW1. RNA concentration and integrity was determined by analysing on a bioanalyzer (Agilent, Santa Clara, CA, USA). RNA was determined to be high enough quality if a minimum RIN of 8.5 was obtained.

### Exon arrays

GeneChip Human Exon 1.0 ST Array analysis was performed as per the ‘GeneChip Whole Transcript (WT) Sense Target labelling assay Manual’ (Affymetrix, Santa Clara, CA, USA). The rRNA from 1 ug of total RNA was reduced using a RiboMinus Human/Mouse Transcriptome Isolation Kit (Invitrogen, Carlsbad, CA, USA). The experimental designs for each experimental group and which and how many patient samples were used can be found in Table S18. Controls were sham irradiated for the same length of time as the 10 Gy samples which was about 20′ at room temperature. Note that four controls were used for each dose response and time course experiment.

### Exon array analysis

For this investigation we have analysed the ‘core set’ that is defined by over 228,000 probe set regions (Affymetrix.com). Assessment of array quality was determined using Expression Console (Affymetrix.com). For differential gene expression, all exon arrays were normalized with RMA background correction and quantile normalization, and then overall transcript expression estimated using Exon RMA linear model [52]. LIMMA [53] was used to contrast among different dosage to identify genes with differential expression. Genes are considered significant if adjusted p-values [54], [55] are less than 0.05. Standard error bars on gene expression graphs represent standard errors based on least square mean. Additionally, the top 100 genes from each contrast were imported into Ingenuity Pathway Analysis for identifying pathways and functional groups that are significantly associated with the gene lists.

The first method to detect AS was determined using AS ANOVA from Partek Genomics Suite statistical analysis package (Partek, St Louis, MO, USA). Secondary methods used to detect alternatively spliced exons, in order to compare all 0 Gy and 10 Gy samples for LCLs and fibroblasts independently, utilized Splicing Index (ie. log NI scores) [56] and FIRMA [57]. Splicing Index is the official alternative-splicing analysis method for Exon 1.0ST developed by Affymetrix itself. FIRMA is another method targeted at the Exon 1.0ST array platform and is claimed to provide more robust results on a wide range of data sets [57]. Our strategy is to identify genes commonly found by the three methods to reduce false positives, compiling a gene list of confidence.

Two cohorts of samples were designed specifically for the interrogation of time and dose dependent genes. The time-dependent cohort consists of samples at 0, 2, 4, 8 and 24 hours post irradiation (10 Gy). The dose-response cohort consists of samples irradiated with 0, 1, 2, 5, 10 and 20 Gy, taken 4 hours post-irradiation. Time and dose response genes were identified using EDGE, a significance analysis method designed for time course experiments [58], [59]. Natural cubic splines were chosen in all cases for fitting expression profiles over time/dose and false discovery rate (Q-value) threshold of 0.05 was used. Some apoptosis is known to be measurable in LCLs at the later time points (e.g. 24 hr and 48 hr) and this probably contributes to some of the noise observed at the later time points.

### Transcriptional validation

Primers were designed to candidate exons or genes using ‘Primer3’ on-line software [60]. The primers were then checked for secondary structure (Premier Biosoft International) and for uniqueness using NCBI primer blast (ncbi.nih). All primer sequences are shown (Table S1). cDNA was prepared from total RNA using Superscript III as per manufacturer's recommendation (Invitrogen, San Diego, USA). Normal PCR amplification was carried out using 1.25 units of GoTaq polymerase (Promega, Wisconsin, USA), 200 nM primers, 500 ng cDNA, with a cycling protocol of 95°C: 2 min; ((95°C: 15 sec; 60°C, : 45 sec; 72°C: 30 sec)×30); 72°C: 5 min. Three primer pairs a different annealing temperature was used (Table S1) Products were run on a 2% or 4% agarose gel or a 5% polyacrylamide gel to determine amplification of the proper sized product. Real-time PCR was performed using these primers under the following conditions. Power SYBR Green Master Mix (Applied Biosystems, United Kingdom) was mixed with 100 ng of cDNA and 3.2 pmol of each primer. The cycling steps were as follows. 95°C: 10 min; ((95°C: 15 sec; 60°C: 60 sec)×40); with a melting curve temperature ramp following.

### Cloning of PCR amplicons and sequencing

PCR amplicons were cut out of poly acrylamide gels and the DNA was eluted in elution buffer overnight at room temperature. Direct sequencing from PCR amplicons was completed by cutting out the appropriately sized band and purifying the amplicon using a Qiagen gel purification column or by stabbing the band of interest using a pipet tip followed by re-amplification and clean-up using a Qiagen PCR product spin column. In other cases the isolated PCR amplicon was ligated to the pGEMeasy-T linearized vector as per manufacturer's recommendations (Promega). For sequencing from clonal inserts, we utilized the amplicon primers. Big Dye terminator sequencing was performed using T7 and SP6 primers and the splice sites at the nucleotide level were determined by sequence comparison.

### 5′ RNA ligase-mediated rapid amplification of cDNA ends (5′-RLM-RACE)

5′-RLM-RACE was performed using FirstChoice RLM-RACE kit (Ambion, Austin, TX, USA) recommended by the manufacturer except the CIP digested RNA was purified using RNAeasy kit (Qiagen, Venlo, The Netherlands). The MDM2 transcript was amplified using nested PCR (as recommended by Ambion) with forward (inner and outer) primers to the adaptor (provided with the FirstChoice RLM-RACE kit) and reverse (inner and outer) MDM2 specific primers (Table S1).

## Supporting Information

Figure S1 **Gene ontologies that are enriched after IR.** The top 10 gene ontology functional (A, C) and pathway (B, D) categories using the top 100 up- and down-regulated genes 4 hours after 10 Gy in LCLs (A, B) and fibroblasts (C, D). The threshold for significance is indicated (horizontal straight line). The ratio of total number of genes in a gene ontology pathway category divided into the number of genes from the 100 gene input is indicated by the squares.
(TIF)

Figure S2 **Gene networks after IR.** (A) Gene network of IR-modulated genes in LCLs. Genes such as CCNB1 and NFkB complex are central in this network. Solid lines represent direct protein interactions and dashed lines represent indirect interactions. Shaded genes are genes that are in the top 100 up-regulated (red) and top 100 down-regulated (green) genes shown to be modulated at 4 hours following 10 Gy IR in LCLs. (B) Gene network of IR-modulated genes in fibroblast cells. Genes such as CDKN1A and MDM2 are central in this network. Solid lines represent direct protein interactions and dashed lines represent indirect interactions. Different shapes correspond to different gene ontological groupings (Ingenuity Pathway Analysis). Shaded genes are genes that are in the top 100 up-regulated (red) and top 100 down-regulated (green) genes shown to be modulated at 4 hours following 10 Gy IR in fibroblast cells.
(TIF)

Table S1 **PCR primer sequences.**
(XLSX)

Table S2 **Genes modulated 4 hours post 10 Gy IR in LCLs.**
(XLSX)

Table S3 **Genes modulated 4 hours post 10 Gy IR in fibroblast cell lines.**
(XLSX)

Table S4 **Novel IR-modulated genes in LCLs.**
(XLSX)

Table S5 **Novel IR-modulated genes in fibroblasts.**
(XLSX)

Table S6 **Genes predicted to be alternatively spliced in LCLs at 4 hours post 10 Gy IR based on Partek.**
(XLSX)

Table S7 **Genes predicted to be alternatively spliced in LCLs at 4 hours post 10 Gy IR based on FIRMA.**
(XLSX)

Table S8 **Genes predicted to be alternatively spliced in LCLs at 4 hours post 10 Gy IR based on SI.**
(XLSX)

Table S9 **Genes predicted to be alternatively spliced in fibroblasts at 4 hours post 10 Gy IR based on Partek.**
(XLSX)

Table S10 **Genes predicted to be alternatively spliced in fibroblasts at 4 hours post 10 Gy IR based on FIRMA.**
(XLSX)

Table S11 **Genes predicted to be alternatively spliced in fibroblasts at 4 hours post 10 Gy IR based on SI.**
(XLSX)

Table S12 **Dose response in LCLs.**
(XLSX)

Table S13 **Dose response in fibroblasts.**
(XLSX)

Table S14 **Time course in LCLs to 24 hours.**
(XLSX)

Table S15 **Time course in LCLs to 48 hours.**
(XLSX)

Table S16 **Time course in fibroblasts to 24 hours.**
(XLSX)

Table S17 **Time course in fibroblasts to 48 hours.**
(XLSX)

Table S18 **The percentage of genes modulated on individual chromosomes.**
(XLSX)

Table S19 **Identification of the patient derived cell lines used for each experiment.**
(XLSX)
