# Supplementary figures and images for: Alternative Transcript Initiation and Splicing as a Response to DNA Damage
Source: PLoS One. 2011 Oct 19;6(10):e25758. doi: 10.1371/journal.pone.0025758 (PMC3198437; doi:10.1371/journal.pone.0025758)

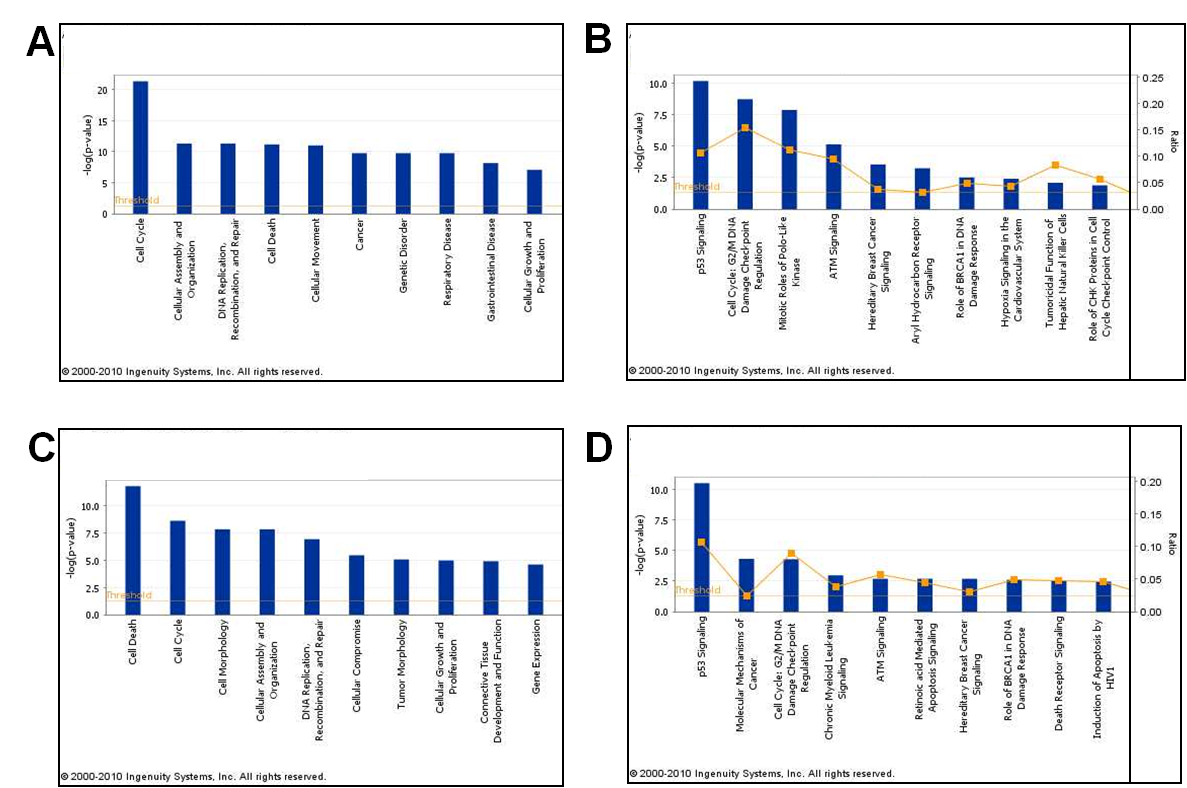

Supplement: Figure S1 — Gene ontologies that are enriched after IR. The top 10 gene ontology functional (A, C) and pathway (B, D) categories using the top 100 up- and down-regulated genes 4 hours after 10 Gy in LCLs (A, B) and fibroblasts (C, D). The threshold for significance is indicated (horizontal straight line). The ratio of total number of genes in a gene ontology pathway category divided into the number of genes from the 100 gene input is indicated by the squares. (TIF) [file pone.0025758.s001.tif]

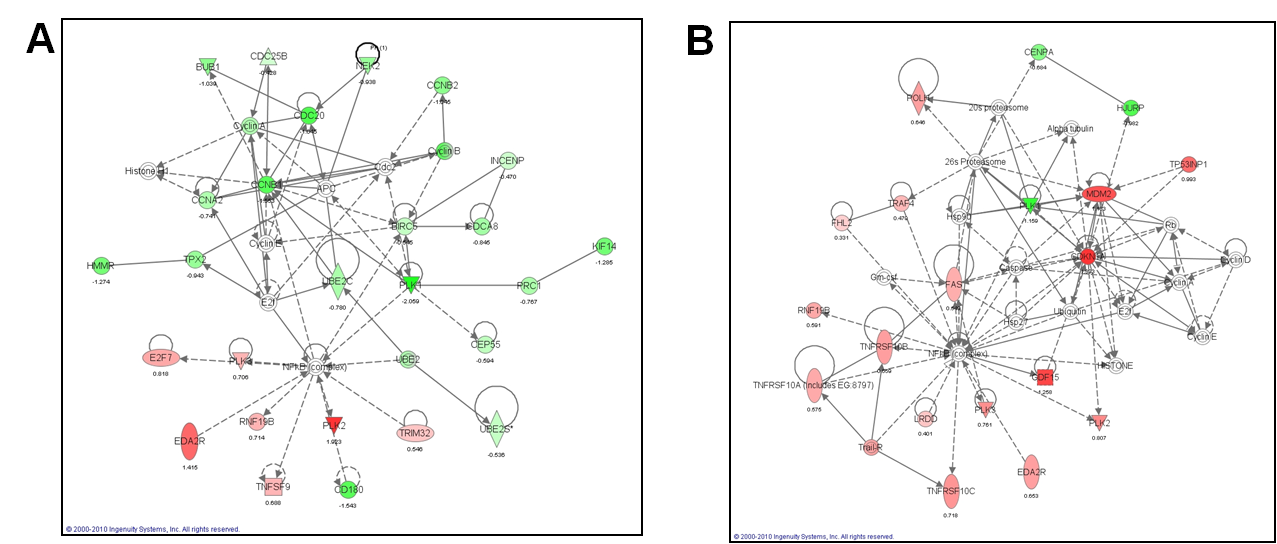

Supplement: Figure S2 — Gene networks after IR. (A) Gene network of IR-modulated genes in LCLs. Genes such as CCNB1 and NFkB complex are central in this network. Solid lines represent direct protein interactions and dashed lines represent indirect interactions. Shaded genes are genes that are in the top 100 up-regulated (red) and top 100 down-regulated (green) genes shown to be modulated at 4 hours following 10 Gy IR in LCLs. (B) Gene network of IR-modulated genes in fibroblast cells. Genes such as CDKN1A and MDM2 are central in this network. Solid lines represent direct protein interactions and dashed lines represent indirect interactions. Different shapes correspond to different gene ontological groupings (Ingenuity Pathway Analysis). Shaded genes are genes that are in the top 100 up-regulated (red) and top 100 down-regulated (green) genes shown to be modulated at 4 hours following 10 Gy IR in fibroblast cells. (TIF) [file pone.0025758.s002.tif]
